# Inference of reticulate evolutionary histories by maximum likelihood: the performance of information criteria

**DOI:** 10.1186/1471-2105-13-S19-S12

**Published:** 2012-12-19

**Authors:** Hyun Jung Park, Luay Nakhleh

**Affiliations:** 1 Department of Molecular and Cellular Biology, Baylor College of Medicine, Houston, TX, USA; 2 Department of Computer Science, Rice University, Houston, TX, USA

## Abstract

**Background:**

Maximum likelihood has been widely used for over three decades to infer phylogenetic trees from molecular data. When reticulate evolutionary events occur, several genomic regions may have conflicting evolutionary histories, and a phylogenetic network may provide a more adequate model for representing the evolutionary history of the genomes or species. A maximum likelihood (ML) model has been proposed for this case and accounts for both mutation within a genomic region and reticulation across the regions. However, the performance of this model in terms of inferring information about reticulate evolution and properties that affect this performance have not been studied.

**Results:**

In this paper, we study the effect of the evolutionary diameter and height of a reticulation event on its identifiability under ML. We find both of them, particularly the diameter, have a significant effect. Further, we find that the number of genes (which can be generalized to the concept of "non-recombining genomic regions") that are transferred across a reticulation edge affects its detectability. Last but not least, a fundamental challenge with phylogenetic networks is that they allow an arbitrary level of complexity, giving rise to the model selection problem. We investigate the performance of two information criteria, the Akaike Information Criterion (AIC) and the Bayesian Information Criterion (BIC), for addressing this problem. We find that BIC performs well in general for controlling the model complexity and preventing ML from grossly overestimating the number of reticulation events.

**Conclusion:**

Our results demonstrate that BIC provides a good framework for inferring reticulate evolutionary histories. Nevertheless, the results call for caution when interpreting the accuracy of the inference particularly for data sets with particular evolutionary features.

## Introduction

W. Maddison proposed a likelihood framework for inferring species trees by simultaneously accounting for evolutionary events within loci (that is, mutations at the nucleotide level) and across loci (that is, gene tree incongruence) [1]. The post-genomic era has highlighted and further stressed the need for inference under such a framework, as analyses of different data sets have revealed varying degrees of incongruence among gene trees; e.g., [2-7]. All these analyses have focused on *deep coalescence *as the source of gene tree incongruence. Another source of incongruence that has long been acknowledged by biologists and that is being increasingly revealed by phylogenomic analyses is *reticulate*, or, non-treelike, evolutionary events. For example, evidence shows that bacteria may obtain a large proportion of their genetic diversity through the acquisition of sequences from distantly related organisms, via horizontal gene transfer (HGT) [8-15]. Furthermore, evidence of widespread HGT in plants has also emerged [16-18]. Interspecific recombination is believed to be ubiquitous among viruses [19,20]. Hybrid speciation is a major evolutionary mechanism in plants, and it is also seen in groups of fish and frogs [21-26]. Further, hybridization is believed to play an important role in speciation and evolutionary innovation in several groups of plant and animal species [27,28].

When reticulate evolutionary events occur among species, the species phylogeny takes the shape of a *network*, which is a rooted, directed, acyclic graph that extends the evolutionary tree model by incorporating non-vertical inheritance of genetic material [29]. Jin *et al*. [30] restricted the maximum likelihood (ML) framework of [1] to the case where gene tree incongruence is exclusively due to horizontal gene transfer (HGT) events, thus providing a maximum likelihood formulation of the problem of inferring phylogenetic networks from sequence data. While the maximum likelihood (ML) formulation of [30] showed good performance in inferring reticulations on synthetic and biological data sets, it is not clear what parameters affect the performance of ML in general. We hypothesize that the diameter of the reticulate evolutionary event (e.g., the distance between the donor and recipient of an HGT event) plays an important role in the detectability of such an event. Further, as more complex networks (that is, ones with more reticulations) necessarily fit the data better than simpler ones, it is important to address the over-fitting issue [29]. In this paper, we conduct simulation studies to assess the effect of the evolutionary diameter on the identifiability of reticulation events. Further, we investigate the performance of two commonly used information criteria for controlling for the complexity in inferred phylogenetic networks, namely the Akaike Information Criterion (AIC) [31] and the Bayesian Information Criterion (BIC) [32]. These criteria have been used for model selection in molecular phylogenetics and their performance has been assessed [33,34]. Further, these criteria have been used in the context of phylogenetic networks recently to distinguish between reticulation events and incomplete lineage sorting [35,36]. However, none of these works studied the performance of these criteria for the problem.

Our results show that, under the conditions we investigate, ML performs well in terms of estimating inheritance probabilities, and less so in determining the location, or placement, of reticulation edges. They also show that the diameter, inheritance probability, and number of gene data sets used combined have a significant effect on the performance. We find that BIC, and to a lesser extent AIC, performs very well in terms of model selection and preventing ML from grossly overestimating the amount of reticulation.

## Methods

### Phylogenetic networks and trees

While the phylogenetic network model is general enough to allow for modeling all types of reticulate evolutionary events, such as hybrid speciation, recombination, and horizontal gene transfer (HGT), the semantics of the model change based on the specific evolutionary events allowed [29]. We focus here on hybridization and HGT as the reticulate evolutionary events, and adopt the following phylogenetic network model. In particular, we exclude events such as *deep coalescence*.

**Definition 1 ***A (binary) *phylogenetic *χ*-network *N is a tuple *(*G*, *f*, *γ*), *where:*

• *G=(V, E) is a rooted, directed, acyclic graph (DAG) with V = V_T _*∪ *V_H _,where V_T _(tree nodes) is the set that contains the root (node r with in-degree 0 and out-degree 2), the set V_L _of leaves (nodes with in-degree 1 and out-degree 0), and the set V_I _of internal nodes other than the root (nodes with in-degree 1 and out-degree 2); V_H _(reticulation nodes) is the set of nodes with in-degree 2 and out-degree 1; and E is the set of the network's edges (we distinguish between the set E_T _of tree edges, whose heads are tree nodes, and the set E_H _of reticulation edges, whose heads are reticulation nodes)*.

• *f *: *V_L _*→*χ is a leaf-labeling bijection*.

• * γ *: *E_H _*→[0,1] *maps the inheritance probabilities to reticulation edges, and satisfies $\gamma \left(e_{1}\right)+\gamma \left(e_{2}\right)=1$ for every pair of edges e*_1 _*and e*_2 _*that share the same reticulation node at their heads*.

As the name implies, the interpretation of *γ *is the probability of inheritance of a gene from each of the two potential parents, and is estimated from the data [30,35-38]. A phylogenetic *χ*-tree is an *χ*-network in which *V_H _*= ∅. While a network *N *represents the evolution of a set of genomes, these genomes can be partitioned into (non-recombining) regions *R_1_,R_2_*, *..*., *R_k_*, each of which has a treelike evolutionary history *T_i_*. In other words, the set $T=\left\{T_{1},...\;,T_{k}\right\}$ is a subset of the set of all trees *contained *within the network *N *(it is worth mentioning that each of these regions can be taken to correspond to a single site in the genomic sequences under study, but our preliminary analyses indicate that such an approach would result in gross overestimation of the amount of reticulation in a data set). More formally, T⊆T(N), where T(N) is the set of *all *trees obtained as follows from *N*: (1) for each node of in-degree 2 remove one of the two incoming edges and (2) for each node *u *of in-degree and out-degree 1, remove *u *along with its incident edges, and add a new edge to connect *u*'s parent to *u*'s child (this step is repeated until no such nodes *u *remain). For a tree T∈T(N), an *induction set *of *T*, denoted by *η*(*T*), is a set of reticulation edges in *N *that are used (that is, not removed in step (1) above) to obtain tree *T*. Notice that *η*(*T*) is not necessarily unique for a given tree *T *as there may be more than one possible way of obtaining tree *T *[39]. Using this framework, we can define the probability of observing a tree *T*, given a phylogenetic network *N*, along with its inheritance probabilities, as $P\left(T\;|\;N,\gamma \right)=\sum_{\eta \left(T\right)\in I(T)}\left[\prod_{e\in \eta \left(T\right)}\gamma \left(e\right)\right]$, where *I*(*T*) is the collection of all induction sets of tree *T*.

### Phylogenetic networks and maximum likelihood

Given a collection *R*_1_,*R*_2_,..., *R_k _*of non-recombining genomic regions (we take them to be "genes" below), and set *S *= {*S*_1_, *S*_2_, ..., *S_k_*}, where *S_i _*is the sequence alignment corresponding to region *R_i_*, the likelihood function, as proposed in [30], is given by

(1) $$L\left(N,\gamma |S\right)=\underset{S_{i}\in S}{\prod }\left[\underset{T\in T\left(N\right)}{\sum }\left[P\left(S_{i}|T\right)\cdot P\left(T|N,\gamma \right)\right]\right],$$

where **P**(*S_i_*|*T*) represents the tree likelihood score, and **P**(*T *|*N*, *γ*) is the probability of observing gene tree *T*, given phylogenetic network *N *and the inheritance probabilities *γ*. The ML framework for inferring reticulation evolutionary histories from a set  S for loci amounts to identifying the phylogenetic network *N *(topology and branch lengths) along with the inheritance probabilities vector *γ *that maximize Eq. (1).

### Information criteria

Given a phylogenetic network *N*, it can be augmented into a phylogenetic network *N'*, by adding further reticulation nodes and edges. By definition of the set of trees contained within a network, we obtain the relationship $T\left(N\right)\subseteq T\left(N^{\prime }\right)$. Using this relationship in conjunction with Eq. (1), we obtain $L\left(N,\gamma \;|\;S\right)\leq L\left(N^{\prime }\;D,\gamma^{\prime }\;|\;S\right)$, where *γ' *is the inheritance probabilities vector associated with phylogenetic network *N' *(with the inheritance probabilities of the reticulation edges that are shared by *N *and *N' *remaining unchanged). In other words, augmenting the network results, in most cases, in a better fit of the data, and never in a worse fit [29]. Based on this observation, a phylogenetic network inference procedure that seeks the network that maximizes Eq. (1) without accounting for network complexity (in terms of the number of reticulation nodes) would produce unrealistic evolutionary histories with large numbers of reticulation events.

To address this issue, we explore in this paper two information criteria, AIC [31] and BIC [32], which are widely used for model selection problems. The AIC criterion is defined as

(2) AIC=2K- 2lnL,

where *K *is the number of parameters in the model, and *L *is the likelihood of the estimated model. BIC [32] measures the balance between goodness-of-fit and the noise based on the following formula:

(3) BIC=Klnn- 2ln L,

where *K *and *L *are defined as above, and *n *is the sample size. When using these criteria, the model with the smallest value is sought. In our context, *K *corresponds to the number of the branches of the network, *L *is given by Eq. (1), and *n *is the total number of sites in all genes in the sequence data set.

### Searching the phylogenetic network space

We implemented a heuristic search procedure that starts from an initial tree *T*, and then searches all networks obtained from *T *by adding a single reticulation node, identifying an optimal network *N*_1_, then all networks obtained from *N*_1 _by adding a single reticulation node, and so on. When analyzing a real data set, *T *is an underlying tree that captures vertical inheritance (e.g., in a study that uses whole-genome data, the majority consensus of all trees on the regions might be a good starting tree *T*). To add a reticulation node to a network (or tree), the procedure picks a pair of edges (*u*_1_, *v*_1_) and (*u*_2_, *v*_2_), subdivides each edge into two edges of equal length (each of the two edges is half the length of the original edge that was subdivided), such that we have (*u*_1_, *x*_1_), (*x*_1_, *v*_1_), (*u*_2_, *x*_2_), and (*x*_2_, *v*_2_), and finally, it adds a reticulation edge between *x*_1 _and *x*_2 _(in either direction). It is important to note that in this procedure, when the pair of edges is picked for adding a reticulation node, cycles are excluded, as well as reticulation edges between two tree edges emanating from the same node ("sibling edges"). In our search procedure, we begin with a tree (the species tree), and then consider networks with higher numbers of reticulation nodes. The set of all networks with *k *+ 1 reticulation nodes is not generated "from scratch" by adding *k *+1 reticulation nodes in all possible ways to the initial tree *T*; rather, it is generated by adding a single reticulation node, in all possible ways, to the optimal network with *k *reticulation nodes. For each number of reticulation nodes, we maintain the network with the optimal value for the information criterion. In other words, we build the network model using forward selection with potential reticulation nodes as variables, rather than an exhaustive model building. Even though the feature selection approach has its own issues, it has been shown to provide good results [30,40]. For each phylogenetic network, we also need to compute the inheritance probabilities *γ *that optimize Eq. (1). For this purpose, we used a grid search with values for each inheritance probability in the set {0.05, 0.1,..., 0.5}. Finally, to compute the probabilities **P**(*S_i_*|*T*) in Eq. (1), we used the dnaml program packaged in Phylip [41] with the K80 model of evolution.

To put it all together, given a phylogenetic network *N *with *h *reticulation nodes, we identify the optimal phylogenetic network *N' *with *h *+ 1 reticulation nodes using the equation

(4) $$\left(e^{*},\gamma^{*}\right)=\text{argma}{\text{x}}_{\left(e,\gamma \right)}L\left(N\text{'},\gamma |S\right),$$

where (*e,γ*) ranges over all possible ways of adding a reticulation edge *e *with inheritance probability *γ *∈ {0.05,0.1, ..., 0.5} to produce phylogenetic network *N' *that differs from *N *by a single reticulation node. Here, the vector *γ *of inheritance probabilities includes those of phylogenetic network *N *and the inheritance probability *γ *of the new reticulation edge *e*. Once the pair (*e*^∗^, *γ*^∗^) is identified, the phylogenetic network *N' *is obtained by adding reticulation edge *e*^∗ ^to *N*, with its inheritance probability *γ*^∗^.

## Results

In this section, we investigate the effects of topological properties of reticulation events on the performance of an ML approach to phylogenetic network inference. Further, we study the performance of ML in terms of estimating the inheritance probabilities from sequence data, and then investigate how the two information criteria perform in terms of estimating the number of reticulation events in a data set. For the synthetic data we analyze here, we used the PhyloGen program [42] to generate species trees under a birth-death model. Each species tree was then used to generate gene trees with HGT events using the tool of [43] (which does not generate deep coalescence). Since Galtier's tool does not give information about the actual HGT events simulated, we modified the tool so that it produces such information. We then used the Seq-gen tool [44] to simulate the evolution of DNA sequence data sets, each of length 100 sites, down each of the gene trees, using the K80 model with transition/transversion ratio of 2 (the sequence at the root was generated randomly by Seq-gen). We describe below the details of the remaining steps of the simulation setup that are specific to each study. It is important to emphasize that we do not conduct exhaustive evaluation of the entire network space, but rather do a heuristic search as described above. While this can have an effect on the results obtained, we believe that under the simulation setup we use here, the results are not affected.

### Effect of the diameter and height of reticulation events

**Figure 1:**
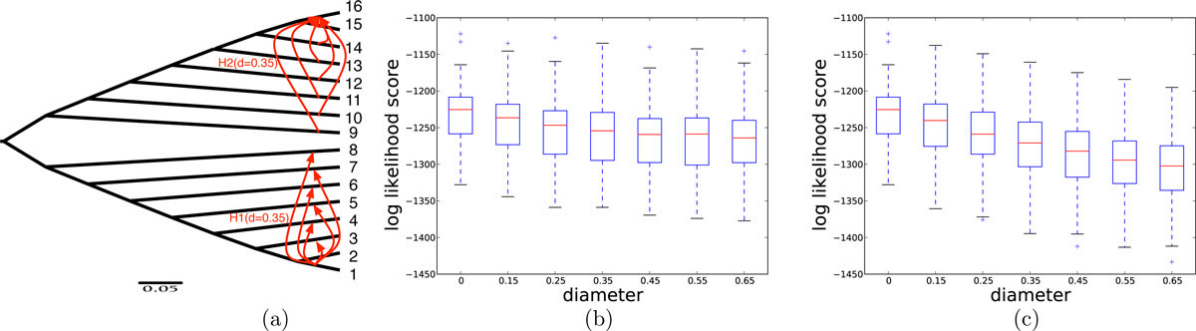
**Effect of the diameter of an HGT edge on the change in the likelihood score**. The diameter of an HGT edge from node *x *to node *y *in the phylogenetic network is measured as the length of the path between *x *and *y *in the underlying tree (the network without the red arrows in (a)). Each of the 12 HGT edges was assessed individually, and never in combinations in this experiment. (b) Effect of the diameter for HGTs with different diameters but with a fixed donor node (taxon 1); these results correspond to each of the 6 HGT edges involving taxa 3--8. The diameters of the HGT edges vary from 0.15, for the HGT edge from taxon 1 to taxon 3, to 0.65, for the HGT edges from taxon 1 to taxon 8, with increments of 0.1. (c) Effect of the diameter for HGTs with different diameters but with a fixed recipient node (taxon 16); these results correspond to each of the 6 HGT edges involving taxa 9--14. The diameters of the HGT edges vary from 0.15, for the HGT edge from taxon 14 to taxon 16, to 0.65, for the HGT edges from taxon 9 to taxon 16, with increments of 0.1. Diameter = 0 corresponds to the underlying tree.

Consider a set  S of *k *independent sequence alignments, each of which evolved down a (species) tree *T*. That is, the evolutionary history of  S is reticulation-free. Now, consider evaluating, under maximum likelihood, a hypothesis that involves a single reticulation event along with its associated probability *γ*; i.e., a phylogenetic network *N *that induces the two trees, *T *and *T'*, where *T' *differs from *T *by the placement of a subtree due to a hypothesized reticulation. Under the maximum likelihood framework, the change in the likelihood of the model is $P\left(S\;|N,\gamma \right)-P\left(S|T\right)=\gamma \left[P\left(S|T^{\prime }\right)-P\left(S|T\right)\right]$. This quantity is non-negative whenever *P*(*S*|*T' *) ≥ *P*(*S*|*T*). That is, under the maximum likelihood framework, if an arbitrary tree *T' *has a higher likelihood than the true tree *T *on which the sequences evolved, the ML framework would end up inferring reticulation events, even though the true evolutionary history is reticulation-free. The question we investigate first is: what factors might affect the performance of ML in this case? We hypothesize the diameter of a reticulation event (that is, the length of the path along the underlying species tree between the donor and recipient nodes) and height (that is, the sum of the lengths of the paths from the donor and recipient nodes to the farthest leaves under them, respectively) play a role in the performance of ML. To investigate this question, we conducted the following experiment. We simulated the evolution of 100 sequence alignments, *S*_1_, *S*_2_,..., *S*_100 _down the 16-taxon tree *T *shown in Fig. 1(a), and then calculated *P*(*S_i_*|*T' *), for 1 ≤ *i *≤ 100, where *T *' is one of the 12 trees that differ from *T *by a single subtree prune and regraft (SPR) move, with varying diameters, as shown with the arrows across the tree *T *in the figure. The results show that as the diameter of a falsely postulated reticulation event increases, the probability of the data on that tree decreases compared to the probability on the true tree. Consequently, if the ML criterion errs in inferring reticulation events, it may introduce reticulation events between very closely related taxa. Or, put differently, reticulation events of very low diameter that are inferred by ML may not be well supported. It is important to note that when the recipient is kept fixed, while changing the donor node to increase diameter (Fig. 1(c)), the effect on the decrease of the model likelihood is more than when the donor node is kept fixed and the recipient node changes (Fig. 1(b)). These results combined show that for small diameters where ML may make wrong inferences, the chances are higher that the error involves the placement of the donor node. In general, and beyond the ML framework, one may have more confidence in inference about the recipient than the donor, since in data sets involving bacteria for example, it is very easy to imagine that the true donor is not sampled in the data set given the challenges with sampling bacterial data and the very large population size.

For our second experiment, we generated data as above, yet scored the probabilities of the sequence data on trees that differ from the true underlying tree in a single reticulation event that varies across trees in terms of its height. Unlike the diameter, the height does not seem to have much of an effect on the probabilities beyond the decrease as compared to the probability of the sequences on the true tree (height 0). Results are omitted due to space limitation.

### Performance of ML in determining the placement and probability of reticulation edges

**Figure 2:**
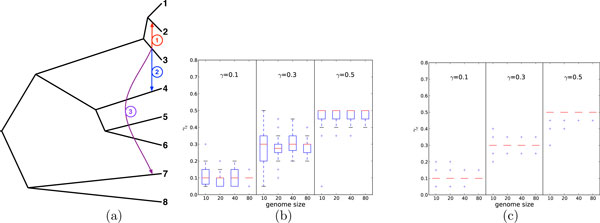
**(a) Three evolutionary histories, each involving the same underlying tree (black lines) and a single reticulation edge from the set of three reticulation edges 1, 2, and 3**. The diameters of the three reticulation edges 1, 2, 3 are 0.5, 1.0, and 1.5, respectively. (b,c) The performance of ML for estimating the inheritance probabilities on data simulated with a single reticulation event. The genome size corresponds to the number of gene data sets used in the inference. Each panel contains three segments, corresponding to three different values of true inheritance probabilities: 0.1, 0.3, and 0.5. The inheritance probabilities *γ_e _*were estimated using Eq. (4). The two diameters of *d *= 0.5 (b) and *d *= 1.5 (c) correspond to the two networks of (a), with HGT edges 1 and 3, respectively; results for the third network are omitted due to space limitations. See text for more details.

In our second set of experiments, we set out to investigate how ML performs in terms of identifying the location of a reticulation edge as well as the inheritance probability that indicates the fraction of genes (non-recombining regions) that were transferred across that edge. We considered three independent evolutionary scenarios, each involving a single reticulation edge of a certain diameter, as shown in Fig. 2(a). All three reticulation edges have the same height and agree on the donor node, yet differ in terms of recipient node, and consequently the diameter. Each of the three resulting networks contains exactly two trees: (1) Network *N*_1_, which is formed by adding only reticulation edge 1 to the underlying tree *T*; this network contains the two trees *T *and *T*_1_, where *T*_1 _differs from *T *only by placing taxon 2 as a sister taxon of 3; (2) Network *N*_2_, which is formed by adding only reticulation edge 2 to the underlying tree *T*; this network contains the two trees *T *and *T*_2_, where *T*_2 _differs from *T *only by placing taxon 4 as a sister taxon of 3; and, (3) Network *N*_3_, which is formed by adding only reticulation edge 3 to the underlying tree *T*; this network contains the two trees *T *and *T*_3_, where *T*_3 _differs from *T *only by placing taxon 7 as a sister taxon of 3.

To answer the two questions, we generated sequence data as follows: For an inheritance probability *γ *associated with the reticulation edge in network *N_i_*, we evolved (1 − *γ*) of the gene sequence alignments down tree *T*, and *γ *of the gene sequence alignments down the tree *T_i_*. In our experiment, we used inheritance probabilities *γ *∈ {0.1, 0.3, 0.5} and "genome size" in {10, 20, 40, 80}. For each combination of parameter values, we generated 50 data sets and performed inference of reticulation edges and their probabilities on all of them.

To investigate how ML performs in terms of estimating the inheritance probability, we fixed all elements of the model and only inferred the inheritance probability. That is, in this part, we assumed knowledge of the correct placement of the reticulation edge, and inferred the value of its associated *γ *using Eq. (4) (in this case, the equation identifies *γ *while *e *is known). The results are shown in Fig. 2.

There are several points to make. The diameter of the reticulation edge has a great effect on the accuracy of the estimated probabilities. For the largest diameter (*d *= 1.5), the ML criterion estimates the correct value of *γ *in almost all 50 cases, regardless of the true value of *γ*. It is important to note, though, that even for this diameter value, increasing the genome size (number of genes) reduces the variance in the estimates. For the smallest diameter, we observe an accurate estimate of the inheritance probability on average, yet with larger variance across the 50 data sets. In this case as well, increasing the number of genes reduces the variance. Further, for larger values of *γ*, the estimates become more accurate in general.

For studying the performance of ML in terms of placing the postulated reticulation edges, we used the data generated as described above along with the underlying (species) tree, as shown in Fig. 2, and inferred a single reticulation edge for each data set, by using Eq. (4) and the network search procedure. Suppose that network *N *with a single reticulation edge was inferred from data generated down network *N_i _*from Fig. 2. Since both networks *N *and *N_i _*have the same underlying (species) tree, checking whether the inferred reticulation edge agrees in terms of placement with the true one is equivalent to checking whether the other tree *T' *(besides *T*) induced by *N *is identical to the tree *T_i _*(the one induced by *N_i _*in addition to *T*). However, rather than returning a 0/1 value, we quantify the Robinson-Foulds distance [45] between *T' *and *T_i_*. The results are summarized in Table 1. A value of 0 in the table indicates correct inference of the placement of the reticulation edge and the larger the value in the table the worse the predicted placement.

**Table 1:** The accuracy of the placement of the inferred reticulation edge in terms of the RF distance [45] between the true and inferred gene trees with a single reticulation event (see text for more details). The genome size corresponds to the number of gene data sets used in the inference. The three diameters correspond to the three networks of Fig. 2.

Diameter	*γ *= 0.1 Genome size					*γ *= 0.3 Genome size					*γ *= 0.5 Genome size			
	10	20	40	80		10	20	40	80		10	20	40	80
0.5	0.6	0	0	0		0	0	0	0		0	0	0	0
1	2.3	2.6	1.2	0.3		1.2	0.1	0	0		0.2	0	0	0
1.5	5.6	5.7	5.6	5.5		5.0	3.6	2.3	1.7		3.0	3.2	1.5	0

The genome size corresponds to the number of gene data sets used in the inference. The three diameters correspond to the three networks of Fig. 2.

The results show a very strong effect of the diameter of the true reticulation event on the postulated placement of the inferred one. Holding the inheritance probability and genome size constant, we observe a significant increase in the error as the diameter increases. For example, when using 10 genes and with inheritance probability of 0.1, the error in the placement of the reticulation event increases from 0.6 for diameter 0.5 to 5.6 for diameter 1.5. The same trend holds across all parameter values. This result indicates that confidence in the placement of an inferred reticulation event based on ML decreases as the diameter of the inferred event increases. On the more positive side, and with the exception of diameter 1.5 and inheritance probability of 0.1, increasing the number of genes drastically improves the accuracy of the placement. It is not surprising that for *γ *= 0.1, the error is high even for a large number of genes, since in this case the signal for reticulation is very low. For example, in the case of 10 genes, the evolutionary history of only a single gene involves the reticulation edges; recovering this edge is very hard in this case.

These results highlight an important issue in detecting reticulations using ML. If reticulation is a hybridization or hybrid speciation event, where a large number of genes may be exchanged or transferred across a reticulation edge (that is, a high value of *γ*), then ML would perform very well in terms of identifying the proportion of genes that were transferred horizontally, as well as the actual location of the reticulation (however, see Discussion section about the issue of incomplete taxon sampling). In the case of horizontal gene transfer in prokaryotes, a very small number of genes (or even a fraction of a gene) may be transferred across a reticulation edge; in this case, not much confidence can be assigned to the placement of the reticulation edge, especially if it has a large evolutionary diameter. However, HGT in microbial evolution seems to occur more often between closely related lineages than between distantly related ones [46].

### Model selection under ML and the performance of information criteria

Now that we have explored the effect of diameter on the performance of ML in terms of estimating the placement of reticulation edges along with their associated probabilities, we turn our attention to a most crucial issue with this model, as well as with phylogenetic networks in general, namely model selection. Here, we will investigate how ML does in estimating the correct number of reticulation edges and how, when augmented with information criteria, it performs. Let us denote by *L*(i) the maximum likelihood score of all phylogenetic networks with *i *reticulation edges for a given data set. Then, the AIC criterion selects a phylogenetic network with *i *reticulation edges over a phylogenetic network with *i *− 1 edges only when (2*K *− 2ln *L*(*i *− 1)) − (2(*K *+ 1) - 2 ln *L*(*i*)) > 0. Simplifying this inequality yields ln *L*(*i*) − ln *L*(*i *− 1) > 1. That is, whenever a network with *i *reticulation edges improves the likelihood score by at least one point, over a phylogenetic network with *i *−1 reticulations, the *ith *edge would be selected under AIC, resulting in a more complex network. This is equivalent to *L*(*i*)/*L*(*i *− 1) >*e*. Similarly, for the BIC, a phylogenetic network with *i *reticulation edges is selected over a phylogenetic network with *i *− 1 reticulation edges whenever (*K *ln *n *- 2 ln *L*(*i *− 1)) − ((*K *+ 1) ln *n *- 2 ln *L*(*i*)) > 0, which is equivalent to ln *L*(*i*) − ln *L*(*i *− 1) > ln *n*/2 or $L\left(i\right)/L\left(i-1\right)>\sqrt{n}$. Based on these thresholds, we use 1 as the penalty term of AIC and ln *n*/2 as the penalty term of BIC (since in the results we show below we explore the difference, rather than ratio, of the likelihood scores). In the experiments we now discuss, we focus on the quantity *L*(*i*) − *L*(*i *− 1) as we add more reticulation edges, and compare it to the AIC and BIC penalty terms.

In our first experiment, we set out to investigate how both criteria perform when the data set has no reticulations. We used an experimental setup as above, where we generated 50 sequence data sets based on the (species) tree of Fig. 2 with genome sizes in {10, 20, 40, 80} genes. We then applied our search procedure to identify the best first, second, third, and fourth reticulation edges to add, and compared the changes in likelihood scores, *L*(*i*) − *L*(*i *− 1) to the penalty terms of both information criteria. Results are omitted due to space limitation. We find that the estimated number of reticulation edges under both criteria is always correct (0), except for a few cases when AIC estimates a single reticulation event. Without either of the two criteria, the likelihood improvement is positive whenever any of the four reticulation edges are added. In other words, when no reticulations have occurred, both criteria, and particularly BIC, do a very good job at model selection, whereas ML with no penalty term would grossly overestimate the amount of reticulation.

We now turn our attention to the case of a single reticulation, yet with three different diameters and three different inheritance probabilities, as shown in Fig. 2. The results are omitted due to space limitation. The data used here is the same that we used to obtain the results in Fig. 2 and Table 1 above. The results highlight several issues. For a very small diameter, the change in the likelihood score always exceeds the penalty term of AIC and is always smaller than that of BIC, resulting in accurate estimates based on BIC and overestimates based on AIC. As the diameter increases, to 1, BIC has a very good performance for the larger inheritance probabilities, but underestimates for the case of *γ *= 0.1. However, in this case, increasing the number of genes used to 40 or 80 gives BIC the necessary signal to make an accurate estimation. In the case of a diameter of 1.5, BIC almost always incorrectly predicts 0 reticulations, except when 80 genes are used and *γ *= 0.5. Unlike BIC, AIC performs better at higher diameters, but that is an artifact of the likelihood scores becoming smaller.

These results, combined with the analysis above, indicate that inspecting both the change in the likelihood score itself, as well as the information criteria value may be valuable in determining, for real data sets, the true number of reticulations. An important trend to notice also is that the improvement in the likelihood score decreases when overestimated reticulations are added. Further, the inheritance probability has a clear effect on the performance: the higher the probability, the higher the improvement of the likelihood score becomes, especially as compared to the improvements when overestimating. This again points to the conclusion that it is easier to detect hybridization or hybrid speciation events, where many genes support a reticulation edge, than horizontal gene transfer events involving very small number of genes.

### Results on a biological data set

**Figure 3:**
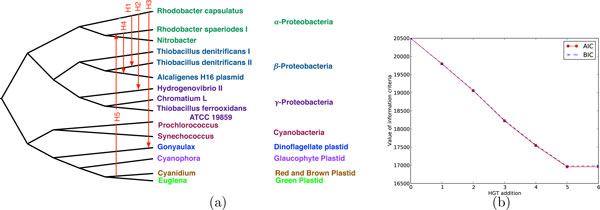
**Results on the *rbcL *gene data set**. (Left) The underlying species tree, as reported in [47], with the five predicted HGT edges posited between pairs of its branches. (Right) The decrease in the AIC and BIC values as optimal HGT edges are added to the species tree. The decrease in the AIC/BIC values from HGT addition *i *to *i *+ 1 corresponds to HGT edge H*i*.

Unlike synthetic data, where the full evolutionary history is known, biological data sets pose several challenges, including the often unknown evolutionary history. In this section, we analyze a 15-taxon dataset of plastids, cyanobacteria, and proteobacteria, which is a subset of the dataset considered by [47] and for which multiple HGT events were conjectured by the authors. For this dataset, we obtained the species (organismal) tree from [47]. The species tree is based on 16S rRNA and other evidence and is shown in Fig. 3. We analyzed the rubisco gene rbcL of these 15 organisms. The gene dataset consists of 15 aligned amino acid sequences, each of length 532 (we used *n *= 532 for BIC). Based on both the AIC and BIC criteria, we infer five HGT events, which agree with the hypotheses of [47] as well as the findings under both maximum parsimony and maximum likelihood analyses of [48] and [30], respectively. The two curves in the figure look very similar simply since the difference between the two terms 2*K *and *K *ln *n *is not visible compared to the large log likelihood values. A major difference between this analysis and the previous computational analyses is that the information criteria systematically determined the number of HGT edges (Fig. 3), whereas in the other analyses the number was determined by an *ad hoc *inspection of the trends of the maximum parsimony and maximum likelihood scores. It is important to mention that in this analysis, we did not infer the inheritance probabilities, but rather set them to 0.5, since only one gene data set was used and estimating the probabilities is not possible from such a data set.

## Discussion

In this paper, we studied the performance of ML for identifying reticulation events from sequence data, based on the formulation given in Eq. (1). We showed through simulation studies that the evolutionary diameter, and to a lesser extent, the height of a reticulation edge affects the performance in terms of estimating the inheritance probability (which reflects the proportion of genes transferred across a reticulation edge) and postulating a placement for the reticulation edge. We showed that increasing the number of genes improves the performance as well. We then investigated the performance of two information criteria, AIC and BIC, and found that BIC in general performs well in terms of model selection and preventing ML from overestimating the number of reticulation edges. Both AIC and BIC produced reasonable results on a biological data set. In this paper, we simulated data on "caterpillar" trees. We will conduct analyses that use other tree shapes to study whether the results hold there as well.

It is important to stress again that the framework, as given by Eq. (1), that we investigated here assumes reticulation as the only source of heterogeneity in the evolution of the sequence data. However, in practice, other events may take place and the model needs to be modified accordingly. In particular, if events such as *deep coalescence *were allowed in the model, then the evolutionary history of a genomic region may take the form of a tree that is not in the set T(N) as we defined it above. Rather, every possible tree topology can now appear in the set T(N), and the probability of each tree can be assessed under models such as the coalescent. Work on accounting for both reticulation and *deep coalescence *simultaneously is emerging [35-38], but dealing with it is beyond the scope of this paper.

Another issue that is of great significance when dealing with reticulation is taxon sampling. As we showed above, the location of the donor node has a significant impact on the detectability of a reticulation edge. When analyzing data sets in practice, particularly prokaryotic data, it may easily be the case that the true donor of the horizontally transferred is not in the data set being analyzed. Therefore, beyond our findings here about the power of ML to infer the placement of a reticulation edge, one has to be cautious about interpreting the placement of a computationally inferred reticulation edge.

A third issue is that while the term reticulation encompasses all types of evolutionary events that are not vertical, there is a clear distinction between, for example, the exchange of a genomic regions through homologous recombination in bacteria and a hybrid speciation event that gives rise to a new species in plants. The amount of genetic material transferred across a reticulation edge in the latter case is much larger than that of in the former. In a phylogenomic study involving thousands of gene families, identifying a reticulation edge that might have been used in the transfer of a single gene might be confounded by the overwhelming vertical signal supported by the remaining genes. Consequently, more confidence can be associated with inferences in cases where a large number of genes support a reticulation edge.

When gene trees are estimated with confidence, one can replace Eq. (1) by $L\left(N,\gamma |T\right)=\prod_{T_{i}\in T}P\left(T_{i}|N,\gamma \right)$, where *T_i _*is the gene tree for gene *i*. In this case, a method for estimating the term **P**(*T_i _| N, γ*) is required. [36] recently devised such a method. We identify comparing this approach to the one we used here as a future research task. Further, in the work of [36], the authors also gave a method to account for uncertainty in the estimated gene trees in set  T, which we will explore as well. We will also compare this approach to the Bayesian approach of [49-51].

Finally, we showed in this manuscript that if the improvement ratio in the likelihood score by adding a reticulation edge is beyond *e *and $\sqrt{n\;}$ for AIC and BIC, respectively, then adding the reticulation edge would be supported. This result can be further pursued in two directions. First, mathematical results can be derived, for specific models of sequence evolution, to establish analytically conditions under which ML would support a reticulation edge, and equivalently, when AIC and BIC would result in overestimation. Second, these results can be utilized for devising efficient algorithmic techniques for identifying reticulation edges whose addition results in significant improvement, as opposed to exhaustively searching the space of all possible reticulation edges, which is infeasible for large numbers of taxa.

## Competing interests

The authors declare that they have no competing interests.

## Authors' contributions

All authors contributed equally.
